# The Efficacy and Adverse Effects of Sugammadex and Neostigmine in Reversing Neuromuscular Blockade Inpatients with Obesity Undergoing Metabolic and Bariatric Surgery: A Systematic Review with Meta-Analysis and Trial Sequential Analysis

**DOI:** 10.3390/medicina60111842

**Published:** 2024-11-08

**Authors:** Shuangwen Wang, Yanjie Dong, Shuangcheng Wang, Yang Han, Qian Li

**Affiliations:** 1 Department of Anesthesiology, West China Hospital, Sichuan University, Chengdu 610041, China; 2 State Key Laboratory of Oral Diseases, National Clinical Research Center for Oral Diseases, West China Hospital of Stomatology, Sichuan University, Chengdu 610041, China

**Keywords:** sugammadex, reversal of neuromuscular blockade, metabolic and bariatric surgery

## Abstract

*Background and Objectives*: Metabolic and bariatric surgery (MBS) is practiced worldwide. Sugammadex was proven to have multiple benefits in reversing neuromuscular blockade (NMB) for patients with obesity undergoing MBS, but its effects on complications of various systems are not clear and concrete. *Materials and Methods:* This systematic review and meta-analysis was conducted as per the PRISMA guidelines and registered on the PROSPERO database (CRD42023491171). A systematic search was conducted in multiple databases for studies comparing sugammadex with neostigmine in MBS. Continuous data are reported as mean differences (MDs) and 95% confidence intervals (CIs). Dichotomous data are reported as relative risks (RRs) and 95% CIs. A two-sided *p* < 0.05 was considered statistically significant. Trial sequential analysis (TSA) was performed to evaluate the reliability of the conclusions. *Results:* Nine studies with 633 patients met the inclusion criteria. Compared with those from the neostigmine group, patients from the sugammadex group were characterized by a significantly shorter recovery time from the administration of the study drug to a train-of-four (TOF) ratio of ≥90% (MD [95% CI]: −15.40 [−26.64; −4.15]; I^2^ = 96.6%; *p* = 0.0073; n = 380; random effects model), a lower risk of postoperative residual curarization (PORC) (RR [95% CI]: 0.18 [0.09; 0.38]; *p* < 0.0001; I^2^ = 27.9%; n = 344; common effect model), postoperative nausea and vomiting (PONV) (RR [95% CI]: 0.67 [0.48; 0.93]; *p* = 0.0164; I^2^ = 0%; n = 335; common effect model), and cardiovascular complications (RR [95% CI]: 0.48 [0.26; 0.88]; *p* = 0.0186; I^2^ = 14.7%; n = 178; common effect model). TSA confirmed the conclusions regarding the recovery time and PORC risk. *Conclusions:* In conclusion, our systemic review and meta-analysis with TSA revealed that sugammadex provided a faster and more reliable choice to reverse NMB in patients with obesity undergoing MBS, with a lower risk of PORC. Sugammadex reduced the risk of cardiovascular complications and postoperative nausea and vomiting. However, the conclusions were not confirmed, and, so, further studies may be necessary.

## 1. Introduction

Metabolic and bariatric surgery (MBS) is an effective and safe treatment for obesity recommended for patients with a body mass index (BMI) of ≥35 kg/m^2^, regardless of co-morbidities [1], and has become one of the most common operations in general surgery. However, performing general anesthesia in patients with obesity comes with challenges. Besides non-ideal airway conditions leading to difficult mask ventilation and intubation [2], typical functional pulmonary changes might develop due to obesity [3,4], including less compliance and reduced residual capacity, possibly contributing to postoperative respiratory complications. Postoperative nausea and vomiting (PONV) is another common complication following bariatric surgical procedures, especially in laparoscopic bariatric surgery (LBS), with a prevalence of up to 65% [5]. Patients with obesity are also prone to postoperative cardiovascular complications due to their increased blood volume, increased cardiac output, and systemic hypertension [6].

Postoperative residual curarization (PORC) is associated with a higher risk of critical respiratory events and affects 60% of patients undergoing general anesthesia [7]. As such, a complete recovery of neuromuscular function after general anesthesia is essential, especially for patients with obesity due to their borderline airway functions. However, when reversing neuromuscular blockade (NMB) in patients with obesity using neostigmine—whose mechanism of action is to increase the concentration of acetylcholine in the synaptic gap—muscle relaxants stored in fat tissue can re-enter the bloodstream after the antidote is eliminated [8], which might increase the risk of prolonged recovery from NMB and PORC. Additionally, PONV and postoperative cardiovascular complications might be aggravated by the use of neostigmine due to its side effects, including glandular secretion, sinus bradycardia, and an emetic effect [9,10].

Sugammadex, a modified γ-cyclodextrin, is a relatively novel drug used to reverse NMB [11]. It allows for fast and complete recovery of neuromuscular function with fewer side effects [12] by binding with the muscle relaxants at the neuromuscular junction, transporting them to the plasma, and being excreted via the kidneys [13]. Among patients with obesity, a systematic review and meta-analysis concluded that the time to reach a train-of-four (TOF) ratio of 0.9 was significantly shorter in patients receiving sugammadex compared with those receiving neostigmine. Furthermore, fewer cases of PORC and other composite adverse events were observed [14], proving its high efficacy and safety.

However, sugammadex might not be the optimal option from a pharmacoeconomic viewpoint. Studies have shown that the high efficacy and safety associated with sugammadex might compensate for its high cost, but solid evidence remains lacking in terms of confirming its efficacy and lowering the risk of PORC. Additionally, it remains controversial whether sugammadex can reduce the incidence of PONV and postoperative cardiovascular complications among patients with obesity undergoing MBS [7,8,15,16]. Therefore, this systematic review with meta-analysis and trial sequential analysis (TSA) was conducted to confirm the efficacy of sugammadex compared with neostigmine when reversing NMB and compare the incidence of adverse effects involving the respiratory, cardiovascular, and digestive systems.

## 2. Methods

This systematic review was carried out strictly per the preferred reporting items for systematic reviews and meta-analyses (PRISMA) guidelines [17] and registered on the PROSPERO database (CRD42023491171). The aim was to compare sugammadex with neostigmine in terms of reversing NMB in bariatric surgery among patients with obesity (age > 18 years and BMI ≥ 35 kg/m^2^), with outcomes defined as the recovery time from administration of the study drug to a TOF ratio of ≥ 90%, the risk of PORC, and so on.

### 2.1. Search Strategy

The Cochrane Central Register of Controlled Trials (Ovid, October 2023), Embase (Ovid, 1974 to 29 November 2023), and PubMed databases were systematically searched, with an updated search on 1 December 2023. Three Chinese databases were also searched, with an updated search on 1 December 2023, including China National Knowledge Infrastructure (CNKI), the China Science and Technology Journal Database, and Wanfang Data. The clinical registry (clinicaltrials.gov) was searched for any missing studies. The specific search strategy is provided in the Supplementary Files.

Two authors (Shuangwen W. and Shuangcheng W.) independently inspected the list of titles and abstracts to identify reports assessed for eligibility. After the full texts of screened articles were retrieved, two investigators (Shuangwen W. and Y.H.) independently assessed their eligibility according to the inclusion and exclusion criteria. Any disagreement was discussed with a third coauthor (Y.D.) and resolved by consensus.

### 2.2. Selection Criteria

The authors reviewed the titles and abstracts of all articles and selected studies that met the following criteria: (1) randomized controlled trials (RCTs); (2) compared sugammadex with neostigmine to reverse NMB in bariatric surgery among patients with obesity (age > 18 years and BMI ≥ 35 kg/m^2^). Conference abstracts meeting the inclusion criteria were also considered as qualified. Studies whose full texts or statistics were not available online were excluded.

### 2.3. Outcome Definition

Our primary outcome was the recovery time from the administration of the study drug to a TOF ratio of ≥ 90%, which was measured in minutes (min). The depth of NMB at the drug's admission could be either moderate (at the second twitch of the TOF) or deep (with a post-tetanic count of 1–5).

The secondary outcomes were the post-anesthesia care unit (PACU) duration (min), TOF at the admission to the PACU, risk of PORC, PONV, and postoperative cardiovascular complications, including bradycardia, hypertension, hypotension, and so on.

### 2.4. Data Extraction

Study characteristics (author, year, type of study) and the demographic details of patients (BMI scale, the type of surgery, the number of patients in each group, the muscle relaxant, the intensity of block at reversal, the sugammadex dose, and the comparison dose) were extracted. Perioperative outcomes were extracted, including time to reach TOF 90%, TOF at PACU, the incidence of PORC, the incidence of PONV, the incidence of postoperative cardiovascular events, and the duration of PACU stay. The extracted data were gathered using WPS Office (V12.2.0.13359).

The data extraction was completed independently by two reviewers (Shuangwen W. and Shuangcheng W.). A third coauthor (Y.D.) was consulted in case of any disagreement.

### 2.5. Quality Assessment

For the quality assessment, two authors (Shuangwen W. and Shuangcheng W.) independently evaluated the potential sources of bias using the Cochrane Collaboration risk-of-bias (RoB2) assessment tool (randomization process, deviations from the intended interventions, missing outcome data, measurement of the outcome, and selection of the reported result) [18]. Each domain was assessed separately and graded as having a ‘low risk’, ‘some concerns’, or ‘high risk’ of bias. The risk of bias (RoB) graphic was created using the RoB2 tool (Cochrane Collaboration). A third author (Y.D.) was consulted in case of conflict. If a trial was assessed as having a high risk of bias, it was excluded from the final meta-analysis.

### 2.6. Statistical Analysis

Means and standard deviations (SDs) were used for continuous variables. All medians, interquartile ranges, and extreme values were converted to means and SDs using an online platform (https://www.math.hkbu.edu.hk/~tongt/papers/median2mean.html, accessed on 31 October 2024), based on previous research [19,20]. A study was excluded from the meta-analysis if the study data were skewed. The numbers of events as a proportion of the sample size were collected for dichotomous variables. Statistical analysis was performed using R studio.

Pooled estimates were obtained using the means and SDs for continuous variables and event rates for dichotomous variables. Continuous data are reported as mean differences (MDs) and 95% confidence intervals (CIs). Dichotomous data were reported as relative risks (RRs) and 95% CIs. A two-sided *p* < 0.05 was considered statistically significant. The χ^2^ test was used to test for the extent of interstudy heterogeneity. The I^2^ statistic was used to describe the proportion of interstudy variation caused by heterogeneity, with an I^2^ value of >50% and *p* < 0.05 representing significant heterogeneity, wherein a random effects model was used to account for the interstudy heterogeneity and increase the robustness of the analysis. Otherwise, a fixed effect/common effect model was adopted. In cases of significant heterogeneity, an influence analysis was performed by excluding studies contributing to the heterogeneity, and the pooled estimates were recalculated. Egger’s test was performed to assess publication bias. If the meta-analysis included trials characterized by both open and laparoscopic surgery, a subgroup analysis including patients undergoing laparoscopic bariatric surgery was performed for each outcome. If data were missing at random, complete-cases meta-analysis was employed, wherein all patients who completed the study were included.

The risk of random errors might increase for both types I and II due to repeated updates of meta-analysis with new RCTs [21], probably leading to exaggerated effects of intervention and spurious results [22,23,24]. TSA is a methodology to evaluate whether the conclusion of a meta-analysis is confirmed and no more trials are needed. In our meta-analysis, TSA was performed using trial sequential analysis software (version 0.9 Copenhagen Trial Unit, Copenhagen, Denmark). The required information size (RIS) was calculated, allowing for a type 1 error of 0.05 and a type 2 error of 0.20. The boundary type was two-sided. For dichotomous data, the relative risk reduction (RRR) was defined as the difference between one and the pooled estimate of the RR, and a control rate was calculated from the unweighted mean of the event proportions in all control groups of the included trials. For continuous data, the MD and variance needed for the TSA boundaries were empirically generated by the software [25]. The robustness of the results usually depends on a sufficient sample size and the accumulation of data. Therefore, TSA was not performed if the number of included trials was fewer than three for a specific outcome to ensure the reliability of the analysis results.

## 3. Results

**Figure 1:**
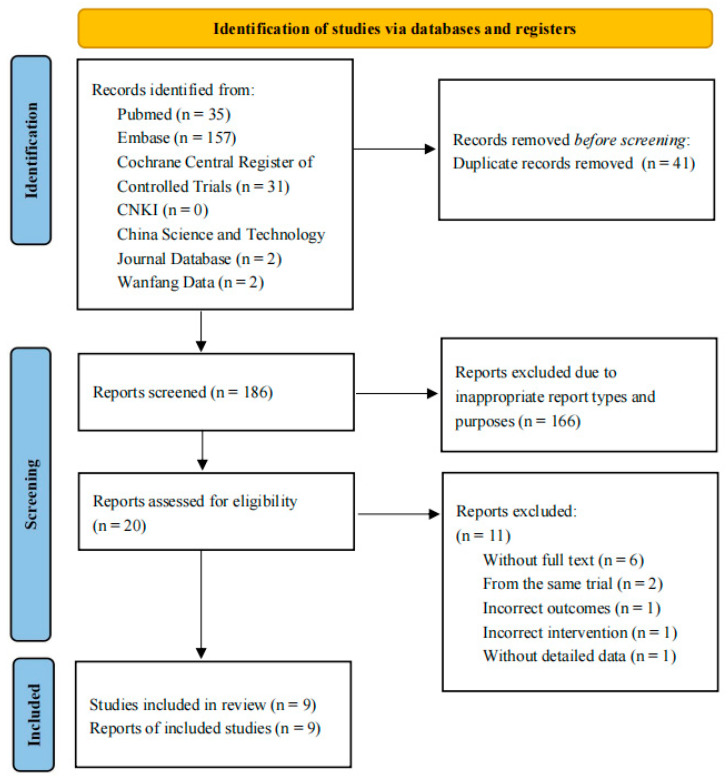
Preferred reporting items for systematic reviews and meta-analyses (PRISMA) flow diagram.

The initial research identified 186 studies to be screened by titles and abstracts, and 20 studies were considered eligible for full-text review. Nine studies with 633 patients (321 received sugammadex and 312 received neostigmine) met the inclusion criteria and were included in the systematic review (Figure 1) [7,8,16,26,27,28,29,30,31], including six RCTs (one of which was published in Chinese) [7,8,16,26,27] and three conference abstracts [28,29,30].

The sample size of the included trials ranged from 28 to 179 adults. The included patients all had morbid obesity with BMIs of >35 kg/m^2^ and underwent various types of bariatric surgery, mostly laparoscopic (77.8%; 7/9). During the surgery, rocuronium was used as the neuromuscular blocking agent at 0.6–1.0 mg/kg, and sugammadex or neostigmine was used as the reversal agent at 2–4 mg/kg or 0.04–0.07 mg/kg, respectively. The doses of these agents were calculated based on different types of weights. The intensity of NMB at reversal was moderate for all trials, except for one study in which it was deep (Supplementary Material S4) [16].

**Figure 2:**
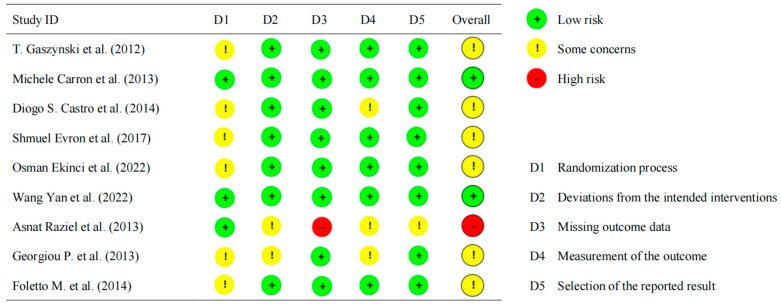
Risk-of-bias (RoB) graphic [7,8,16,26,27,28,29,30,31].

The quality of the studies was assessed using the Cochrane Collaboration risk-of-bias (RoB2) tool. There were some concerns of bias for most trials, mainly arising from the randomization process (Figure 2).

The baseline characteristics were comparable between the intervention and control groups in each trial. Moreover, there was no difference in the mean age (*p* = 0.4217) and BMI (*p* = 0.2591) between the sugammadex and neostigmine groups among patients in the meta-analysis.

**Figure 3:**
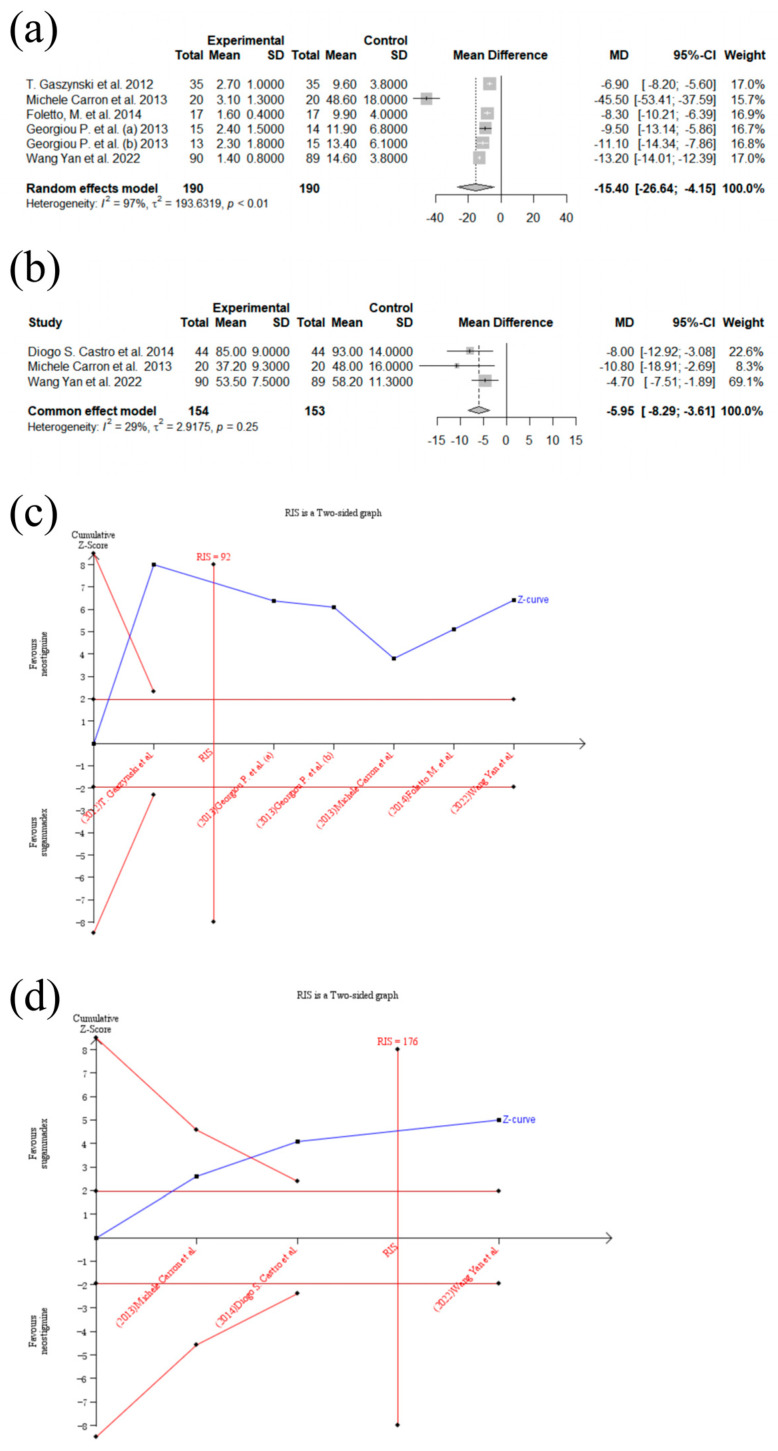
Forest plots and trial sequential analysis of recovery time (min) from the administration of study drug to train-of-four (TOF) ratio ≥ 90% and post-anesthesia care unit (PACU) duration (min). (**a**): Forest plot of recovery time (min) from the administration of study drug to TOF ratio ≥ 90%; sugammadex (experimental) vs. neostigmine (control). (**b**): Forest plot of PACU duration (min); sugammadex (experimental) vs. neostigmine (control). (**c**): TSA of recovery time (min) from administration of study drug to TOF ratio ≥ 90%; sugammadex (experimental) vs. neostigmine (control). (**d**): TSA of PACU duration (min); sugammadex (experimental) vs. neostigmine (control) [7,16,27,28,30,31].

Six trials [7,8,16,28,30,31] reported the primary outcome of the meta-analysis, the recovery time (min) from the administration of the study drug to a TOF ratio of ≥90%. One study was excluded due to skewed data [8], and, so, five trials were included in the meta-analysis. Compared with the control group, the recovery time from the administration of the study drug to a TOF ratio of ≥ 90% was significantly shorter among patients in the sugammadex group (MD [95% CI]: −15.40 [−26.64; −4.15]; I^2^ = 96.6%; *p* = 0.0073; n = 380; random effects model (Figure 3a)). Among the included trials, excluding studies contributing to heterogeneity did not decrease the I^2^ value. However, omitting the trials by Michele Carron et al. and Wang Yan et al. showed an unchanged result with decreased heterogeneity (MD [95% CI]: −8.48 [−10.25; −6.71]; I^2^ = 56.6%; *p* < 0.0001; n = 161; random effects model). The publication bias was not significant (Egger’s test; *p* = 0.7747). In the TSA, the Z-curve crossed the conventional boundary and the sequential monitoring boundary (Figure 3c). It could be assumed that the conclusion was confirmed and no more trials were needed. A subgroup analysis including patients undergoing laparoscopic bariatric surgery was performed, with a contrary result (MD [95% CI]: −22.03 [−44.61; 0.54]; I^2^ = 98%; *p* = 0.0557; n = 253; random effects model; Supplementary Material S5 and Supplementary Figure S1).

Three trials were included in the meta-analysis of the PACU duration [16,27,31], which was significantly shorter among patients receiving sugammadex compared with neostigmine (MD [95% CI]: −5.95 [−8.29; −3.61]; *p* < 0.0001; I^2^ = 28.6%; n = 307; common effect model; Figure 3b). The publication bias was not significant (Egger’s test; *p* = 0.8587). The Z-curve crossed the conventional boundary and the sequential monitoring boundary; thus, the TSA indicated firm evidence in favor of sugammadex (Figure 3d). A subgroup analysis including patients undergoing laparoscopic bariatric surgery was performed, with an unchanged result (MD [95% CI]: −5.95 [−8.29; −3.61]; I^2^ = 28.6%; *p* < 0.0001; n = 307; common effect model; Supplementary Material S5 and Supplementary Figure S2).

**Figure 4:**
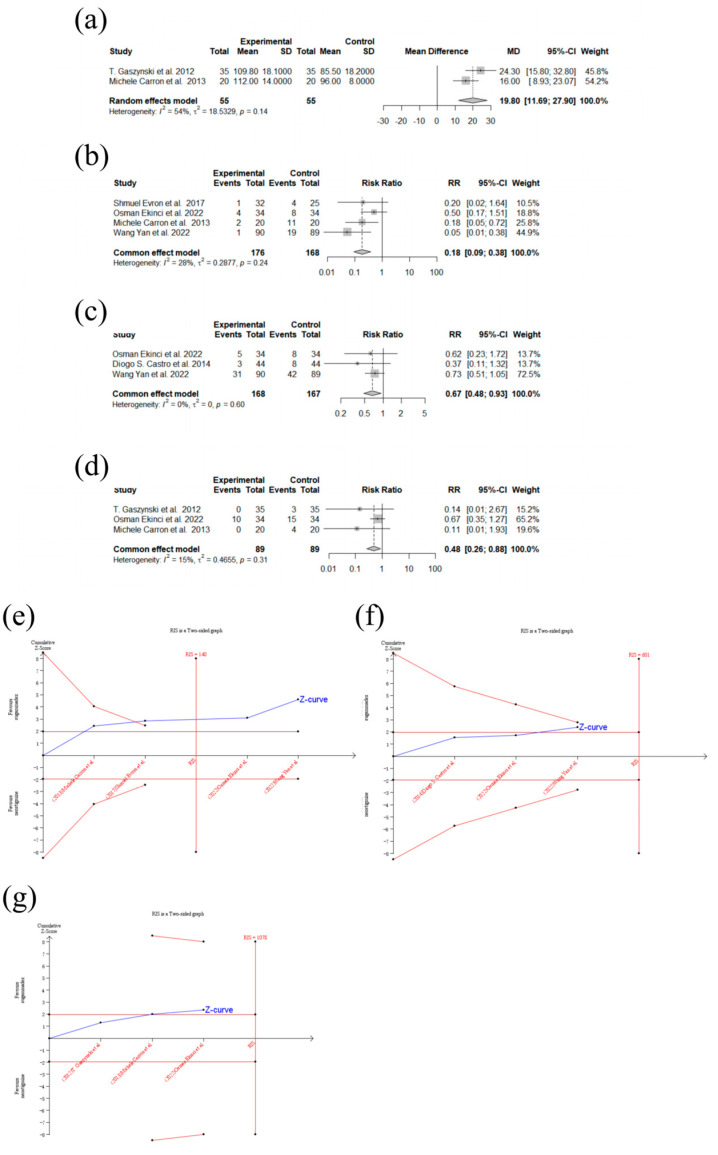
Forest plots and TSA of TOF ratio at admission to PACU, incidence of postoperative residual curarization (PORC), incidence of postoperative nausea and vomiting (PONV), and incidence of postoperative cardiovascular complications. (**a**): Forest plot of TOF ratio at admission to PACU; sugammadex (experimental) vs. neostigmine (control). (**b**): Forest plot of incidence of PORC; sugammadex (experimental) vs. neostigmine (control). (**c**): Forest plot of incidence of PONV; sugammadex (experimental) vs. neostigmine (control). (**d**): Forest plot of incidence of postoperative cardiovascular complications; sugammadex (experimental) vs. neostigmine (control). (**e**): TSA of incidence of PORC; sugammadex (experimental) vs. neostigmine (control). (**f**): TSA of incidence of PONV; sugammadex (experimental) vs. neostigmine (control). (**g**): TSA of incidence of postoperative cardiovascular complications; sugammadex (experimental) vs. neostigmine (control) [7,8,16,26,27,31].

Two trials were included in the meta-analysis of the TOF ratio at admission to the PACU [7,16], which was significantly higher among patients in the sugammadex group than the control group (MD [95% CI]: 19.80 [11.69; 27.90]; *p* < 0.0001; I^2^ = 53.8%; n = 110; random effects model; Figure 4a). Egger’s test and TSA were not performed due to the minimal number of trials [32].

Four trials reported events of PORC (Table 1) and were included in the meta-analysis of the PORC risk [8,16,26,31], which was significantly higher in the control group (RR [95% CI]: 0.18 [0.09; 0.38]; *p* < 0.0001; I^2^ = 27.9%; n = 344; common effect model; Figure 4b). The publication bias was not significant (Egger’s test; *p* = 0.2012). The TSA showed that the Z-curve crossed the sequential monitoring boundary and the conventional boundary, indicating firm evidence in favor of sugammadex (Figure 4e).

Three trials were included in the meta-analysis of the incidence of PONV [8,27,31], which was significantly higher in the neostigmine group (RR [95% CI]: 0.67 [0.48; 0.93]; *p* = 0.0164; I^2^ = 0%; n = 335; common effect model; Figure 4c). The publication bias was not significant (Egger’s test; *p* = 0.3062). The TSA showed that the Z-curve did not cross the sequential monitoring boundary and the line of the RIS, indicating that the available data were too sparse to confirm the conclusion and more trials were needed (Figure 4f).

Three trials reported the incidence of postoperative cardiovascular complications (Table 1) [7,8,16] and were included in the meta-analysis, which indicated significantly fewer cardiovascular adverse events in the sugammadex group than the control group (RR [95% CI]: 0.48 [0.26; 0.88]; *p* = 0.0186; I^2^ = 14.7%; n = 178; common effect model; Figure 4d). The publication bias was not significant (Egger’s test; *p* = 0.0605). The TSA showed that the Z-curve merely crossed the conventional boundary, neither crossing the sequential monitoring boundary nor reaching the line of the RIS, indicating that the available data were too sparse to confirm the conclusion and more trials were needed (Figure 4g). A subgroup analysis including patients undergoing laparoscopic bariatric surgery was performed, with a contrary result (RR [95% CI]: 0.54 [0.29; 1.01]; *p* = 0.0530; I^2^ = 30.5%; n = 108; common effect model; Supplementary Material S5 and Supplementary Figure S3).

## 4. Discussion

We searched both English and Chinese databases in our systematic review and meta-analysis, as these two languages are two of the most popular languages in academic writing [33]. TSA was applied to further confirm that sugammadex is faster in reversing nondepolarizing NMB during MBS, with fewer postoperative complications when compared to neostigmine. There were some concerns of bias for most of the included trials, mainly arising from the randomization process, which needs to be improved in future studies to provide high-quality evidence.

Long-term studies have proven MBS to be an effective, durable, and safe treatment of obesity and its co-morbidities, with superior weight loss outcomes and decreased perioperative and overall mortality [34,35,36,37]. Meanwhile, preferable MBS procedures have evolved from laparotomy into minimally invasive approaches, such as laparoscopy [1]. All included patients in our study were patients with morbid obesity with a BMI of >35 kg/m^2^. Seven out of nine included trials were characterized by laparoscopic bariatric surgery, meeting the criteria and current trends for MBS, which indicates the generalizability of our study.

As sugammadex is expensive, lower-dose sugammadex calculated via the IBW or CBW was considered a cost-saving strategy to cope with the financial burden among patients with morbid obesity; however, its dose was recommended to be calculated via the total body weight (TBW) to avoid residual block [38]. It is hard to strongly recommend lower-dose sugammadex as a substitute for the standard dosing method in patients with morbid obesity due to limited high-quality evidence [39,40,41]. Besides reducing the drug dose, costs may be saved elsewhere. Our meta-analysis and TSA confirmed that sugammadex allowed for faster recovery from NMB and shorter PACU stays in patients with obesity undergoing MBS, consistent with previous observational studies [42,43], which is of economic significance for patients with obesity by balancing the antidote's high cost. Faster recovery from NMB and shorter PACU stays may reduce the time in the operation room. Moreover, if medical staff were to use the time saved to boost productivity and raise the number of cases finished within similar working hours, sugammadex would be cost-effective when compared with neostigmine [44]. A recent study comparing the total costs of healthcare between patients receiving sugammadex and neostigmine found that sugammadex was associated with a USD 232 lower direct cost. The reduction was detectable only among patients with lower risk. The cost was USD 620 higher among patients with an American Society of Anesthesiologists (ASA) physical status of ≥ 3 and preoperative hospitalization, which might have resulted from the side effects of sugammadex and other unrelated complications leading to longer hospital stays [45]. Considering the high level of heterogeneity of our meta-analysis, an influence analysis was performed with unchanged results. The trial by Michele Carron et al. was omitted as deep NMB was adopted, while other included trials used moderate NMB. The heterogeneity of the trial by Wang Yan et al. might be attributed to the use of different manufacturer’s drugs and an NMB monitoring device, leading to the differences in the monitoring data.

PORC, defined as a TOF ratio below 90% [46], is associated with an increased risk of pulmonary complications, such as aspiration and lung inflammation [7]. However, some patients may exhibit obvious symptoms despite achieving a TOF ratio of >90%. Therefore, determining the existence of PORC requires both the objective monitoring of the TOF ratio and clinical evidence of adverse effects potentially attributable to the use of neuromuscular blocking agents [47]. In our meta-analysis, we included trials that reported adverse events probably resulting from residual paresis, such as decreased SpO_2_ and requiring a rescue dose of sugammadex after extubation. Patients with obesity are susceptible to critical respiratory events due to their borderline respiratory physiology, especially when they are accompanied by PORC and impaired respiratory muscle strength. PORC is more commonly seen in patients with morbid obesity than those without obesity (33% vs. 26%) [7]. Therefore, recovery of NMB in patients with obesity needs to be treated more cautiously to avoid residual paresis. Our meta-analysis and TSA confirmed that the risk of PORC was significantly lower and the TOF ratio at admission to the PACU was significantly higher in the sugammadex group than the neostigmine group, making sugammadex a reliable choice to reverse NMB in patients with obesity. Besides preventing PORC, sugammadex can also be used to treat symptoms of PORC symptoms [48].

Our meta-analysis found that the risk of PONV was significantly lower in the sugammadex group in patients with morbid obesity undergoing bariatric surgeries. All three included trials were characterized by LBS [8,27,31], a potential risk factor for PONV [49]. Surgical manipulation might affect the gastric vagal nerve, leading to the stimulation of the nucleus of the solitary tract in the hindbrain that triggers vomiting [50]. CO_2_ pneumoperitoneum is another factor contributing to PONV in laparoscopic surgeries by increasing intra-abdominal pressure (IAP) and decreasing intestinal blood flow [51,52,53]. Meanwhile, sugammadex helps to exhale CO_2_ by rapidly and reliably reversing NMB and assisting the recovery of respiratory muscle, thus acting as a protective factor against PONV. In a post hoc analysis, sugammadex was found to significantly reduce the incidence and severity of PONV among patients with obesity scheduled for laparoscopic bariatric surgeries [15], which is consistent with our findings. Deep NMB is necessary for laparoscopic operations to ensure complete muscle relaxation [54], in which circumstance sugammadex is superior to neostigmine because it allows for a faster and more reliable reversal of NMB, regardless of the degree of NMB at reversal [12,55]. Studies showed that deep NMB facilitated the application of low IAP during laparoscopic surgery, and a low IAP was related to a reduced incidence of PONV [51,54]. Therefore, another assumption is that sugammadex could reduce the risk of PONV compared with neostigmine by allowing deeper NMB and lower IAP. However, the TSA showed that more participants were still needed to reach a confirmed conclusion.

Our meta-analysis indicated significantly fewer cardiovascular adverse events in the sugammadex group compared with the control group in patients with obesity, similar to the results in adult patients [12,56]; however, the conclusion was not confirmed via TSA. Patients with obesity are characterized by an increased blood volume and cardiac output, along with systemic hypertension, which may collectively amplify the incidence of postoperative cardiovascular complications [6]. A retrospective cohort study showed that the use of sugammadex was not associated with a lower risk of major adverse cardiovascular events, including cardiac arrest, acute heart failure, or myocardial infarction [57]. More studies are still needed to decide whether using sugammadex reduces postoperative cardiovascular adverse events.

There were several limitations to our study. First, a majority of the included trials had at least some concerns or high risk of bias, which might have led to over- or under-estimation of the intervention effects. Meanwhile, the gray literature, including conference abstracts, was included in the meta-analysis, which might help to reveal the truth. Second, although TSA was performed to confirm the conclusion, it was unable to adjust for the risk of bias, which might affect the reliability of our findings. Furthermore, considering the high heterogeneity of the included trials for certain outcomes, it might be overly optimistic to indicate that the current studies are sufficient. High-quality RCTs are still needed in the future. Additionally, among the identified trials, the drug doses were calculated based on different dosing scalars, including TBW, CBW, and IBW, which might have led to data dispersivity. However, as the baseline characteristics were comparable in all included trials, the influence can be minimized.

## 5. Conclusions

In conclusion, our systemic review and meta-analysis with trial sequential analysis showed that sugammadex provided a faster and more reliable choice to reverse NMB in patients with obesity undergoing metabolic and bariatric surgery, with a lower risk of postoperative residual curarization. Sugammadex reduced the risk of cardiovascular complications and postoperative nausea and vomiting. Nonetheless, the conclusions were not confirmed, and further studies may be necessary.

## Figures and Tables

**Table 1:** Events of postoperative residual curarization (PORC) and postoperative cardiovascular complications in sugammadex and neostigmine groups.

	Sugammadex Event (n)	Neostigmine Event (n)
PORC		
Shmuel Evron et al. [26]	SpO_2_ < 95% (1)	SpO_2_ < 95% (3); reintubation (1)
Osman Ekinci et al. [8]	Respiratory insufficiency (4)	Respiratory insufficiency (8);
Michele Carron et al. [16]	A decrease of >4% of SpO_2_ versus baseline, SpO_2_ > 90% (2)	A decrease of >4% in SpO_2_ versus baseline, SpO_2_ > 90% (8); requiring transitory oxygen supplementation and a rescue dose of sugammadex after extubation (3)
Wang Yan et al. [31]	TOF < 0.9 (1)	TOF < 0.9 (19)
Postoperative cardiovascular complications		
T. Gaszynski et al. [7]	No event	Bradycardia (3)
Osman Ekinci et al. [8]	Hypotension (1) Hypertension (9)	Hypertension (15)
Michele Carron et al. [16]	No event	Bradycardia (heart rate < 50 beats/min) (4)

## Data Availability

All data generated or analyzed during this study are included in this article. Further inquiries can be directed to the corresponding author.

## Supplementary Materials

The following supporting information can be downloaded at https://www.mdpi.com/article/10.3390/medicina60111842/s1. Supplementary Material S1: PRISMA 2020 checklist (reference is cited in the Supplementary Materials). Supplementary Material S2: Search strategy in PubMed, Embase, Cochrane Central Register of Controlled Trials, China National Knowledge Infrastructure, China Science and Technology Journal Database, and Wanfang Data. Supplementary Material S3: List of excluded studies. Supplementary Material S4: Characteristics of included trials. Supplementary Material S5: Subgroup analysis including patients undergoing laparoscopic bariatric surgery.
