# Supplementary material for: The Efficacy and Adverse Effects of Sugammadex and Neostigmine in Reversing Neuromuscular Blockade Inpatients with Obesity Undergoing Metabolic and Bariatric Surgery: A Systematic Review with Meta-Analysis and Trial Sequential Analysis
Source: Medicina (Kaunas). 2024 Nov 8;60(11):1842. doi: 10.3390/medicina60111842 (PMC11596585; doi:10.3390/medicina60111842)
Supplement: Supplementary file 1 [file medicina-60-01842-s001.zip › Supplementary Materials/Supplementary Material S2 search strategy.pdf]

Supplementary file: search strategy

1. Pubmed (with an updated search on 1 December 2023)

Search Strategy	Results
((((((((((((((((Bariatric Surgery) OR (Surgeries, Bariatric)) OR (Surgery, Bariatric)) OR (Metabolic Surgery)) OR (Metabolic Surgeries)) OR (Surgeries, Metabolic)) OR (Surgery, Metabolic)) OR (Bariatric Surgical Procedures)) OR (Bariatric Surgical Procedure)) OR (Procedure, Bariatric Surgical)) OR (Procedures, Bariatric Surgical)) OR (Surgical Procedure, Bariatric)) OR (Surgical Procedures, Bariatric)) OR (Bariatric Surgeries)) OR (Stomach Stapling)) OR (Stapling, Stomach)) OR ("Bariatric Surgery"[Mesh])) AND ((((((Bridion) OR (6-Perdeoxy-6-per(2-carboxyethyl)thio-gamma-cyclodextrin sodium salt)) OR (Sugammadex Sodium)) OR (Org 25969)) OR (Sugammadex)) OR ("Sugammadex"[Mesh]))	35

2. Embase (with an updated search on 1 December 2023)

Search Strategy		Results
1	exp sugammadex/	3231
2	exp bariatric surgery/	67550
3	1 and 2	157

3. The Cochrane Central Register of Controlled Trials (with an updated search on 1 December 2023)

Search Strategy		Results
1	(Sugammadex or Sugammadex Sodium or Org 25969 or Bridion or 361lpm2t56 or erj6x2mxv7).af.	810
2	(bariatric surgery or Procedures, Bariatric Surgical or Bariatric Surgical Procedures or Bariatric Surgical Procedure or Surgical Procedure, Bariatric or Procedure, Bariatric Surgical or Surgical Procedures, Bariatric or Stapling, Stomach or Stomach Stapling or Bariatric Surgeries or Surgery, Metabolic or Surgeries, Bariatric or Metabolic Surgery or Metabolic Surgeries or Surgery, Bariatric or Surgeries, Metabolic).mp. [mp=title, original title, abstract, floating sub-heading word, mesh headings, heading words, keyword]	3252
3	1 and 2	31

4. China National Knowledge Infrastructure (CNKI) (with an updated search on 1 December 2023)

	Results
（篇关摘：舒更葡糖(模糊)）AND（篇关摘：减重手术(模糊)）<br>(Related terms: sugammadex (ambiguous)) AND (Related terms: bariatric surgery (ambiguous))	0

5. China Science and Technology Journal Database (with an updated search on 1 December

2023)

(题名或关键词=舒更葡糖 AND 题名或关键词=减重手术)<br>(Title or keyword = sugammadex AND Title or keyword = bariatric surgery)	2

6. Wanfang Data (with an updated search on 1 December 2023)

全部:(舒更葡糖) and 全部:(减重手术)<br>All: (sugammadex) and All: (bariatric surgery)	2
