# Supplementary material for: The Efficacy and Adverse Effects of Sugammadex and Neostigmine in Reversing Neuromuscular Blockade Inpatients with Obesity Undergoing Metabolic and Bariatric Surgery: A Systematic Review with Meta-Analysis and Trial Sequential Analysis
Source: Medicina (Kaunas). 2024 Nov 8;60(11):1842. doi: 10.3390/medicina60111842 (PMC11596585; doi:10.3390/medicina60111842)
Supplement: Supplementary file 1 [file medicina-60-01842-s001.zip › Supplementary Materials/Supplementary Material S3 List of excluded studies.pdf]

Sources	Title	Year	Reason of exclusion
The Cochrane Central Register of Controlled Trials	CURES: the Effect of Deep Curarisation and Reversal With Sugammadex on Surgical Conditions and Perioperative Morbidity	2012	Without full text
	Pilot Study on the Pharmacodynamics of Sugammadex in Morbidly Obese Patients: reversal of Deep Neuromuscular Blockade	2013	
	Effects of Neuromuscular Block Reversal With Sugammadex vs Neostigmine on Postoperative Respiratory Outcomes After Major Abdominal Surgery	2014	
	Comparison between sugammadex and neostigmine in the recovery of rocuronium-induced neuromuscular blockade and impact on postoperative pulmonary function in patients undergoing laparoscopic sleeve gastrectomy. A double-blind, randomized controlled trial	2015	
	A Study to Compare Neostigmine vs Sugammadex in Length of PACU Stay in Patients Undergoing Sleeve Gastrectomy Surgery	2017	
	Impact of Sugammadex vs. Neostigmine Reversal on Post-Operative Recovery and Complications	2018	
	Comparative Study Between Neostigmine Versus Sugammadex in Obese Patients Undergoing Laparoscopic Surgery	2020	Without detailed data
Embase	Sugammadex allows a fast-track bariatric anaesthesia	2012	From the same trial
	Comparison of two neuromuscular anesthetics reversal in obese patients undergoing bariatric surgery-a prospective study	2014	
	Suggamadex vs. neostigmine for neuromuscular blockade reversal during surgery in the high risk morbidly obese	2014	Incorrect outcomes
Pubmed	Use of sugammadex is associated with reduced incidence and severity of postoperative nausea and vomiting in adult patients with obesity undergoing laparoscopic bariatric surgery: a post-hoc analysis	2023	Incorrect intervention

Supplementary Table 1: List of excluded studies (the ones that were rejected beyond the phase of ‘full text’)
