# Supplementary material for: The Efficacy and Adverse Effects of Sugammadex and Neostigmine in Reversing Neuromuscular Blockade Inpatients with Obesity Undergoing Metabolic and Bariatric Surgery: A Systematic Review with Meta-Analysis and Trial Sequential Analysis
Source: Medicina (Kaunas). 2024 Nov 8;60(11):1842. doi: 10.3390/medicina60111842 (PMC11596585; doi:10.3390/medicina60111842)
Supplement: Supplementary file 1 [file medicina-60-01842-s001.zip › Supplementary Materials/Supplementary Material S4 Characteristics of included trials.pdf]

Author (year)	Type of study	BMI (kg/m^2) SUG/NEO means ± SD	Type of surgery	number of patients SUG/NEO	Dose of Rocuronium	Intensity of NMB at reversal	Dose of SUG	Dose of NEO (ATR)	Funding
T. Gaszynski et al. (2012)	RCT	47.8 ± 5.9/45.5 ± 5.9	Bariatric surgery	35/35	1.0 mg/kg CBW	TOF count ≥ 2	2 mg/kg CBW	0.05 mg/kg CBW (0.02 mg/kg CBW)	Government funds
Michele Carron et al. (2013)	RCT	48.1 ± 7/47.2 ± 7	Laparoscopic bariatric surgery	20/20	0.9mg/kg IBW	PTCs 1-5	4 mg/kg TBW	0.07 mg/kg LBW, total dose≤5 mg (10 μg/kg, total dose≤1 mg)	Departmental funds
Diogo S. Castro et al. (2014)	RCT	41.2 ± 4.7/41.1 ± 3.7	Laparoscopic bariatric surgery	44/44	Not available	TOF count ≥ 2	2 mg/kg CBW	0.05 mg/kg CBW (0.02 mg/kg CBW)	Not available
Shmuel Evron et al. (2017)	RCT	42.0 ± 6.0/44.0 ± 5.0	Laparoscopic bariatric surgery	32/25	0.6 mg/kg	TOF count ≥ 2	2 mg/kg TBW	2.5 mg (1 mg)	Not available
Osman Ekinci et al. (2022)	RCT	45.6 ± 5.3/48 ± 4	Laparoscopic bariatric surgery	34/34	0.6 mg/kg	TOF count ≥ 2	2 mg/kg	0.05 mg/kg (0.02 mg/kg)	No financial support
Wang Yan et al. (2022)	RCT	43.4 ± 3.8/42.7 ± 3.0	Laparoscopic bariatric surgery	90/89	0.9mg/kg CBW	TOF count ≥ 2	2 mg/kg CBW	0.04 mg/kg CBW (0.02 mg/kg CBW)	Not available
Asnat Raziel et al. (2013)*	RCT	44.8 ± 3.8/41.9 ± 4.2	Laparoscopic bariatric surgery	21/19	Not available	TOF count ≥ 2	Not available	Not available	Not available
Georgiou P. et al. (2013) (a)*	RCT	57.5 ± 7.7/57.4 ± 7.4	Open bariatric surgery	15/14	Not available	TOF count ≥ 2	2 mg/kg IBW	0.05 mg/kg IBW (0.02 mg/kg)	Not available
Georgiou P. et al. (2013) (b)*	RCT	56.5 ± 3.8/56.2 ± 6.3	Open bariatric surgery	13/15	Not available	TOF count ≥ 2	2 mg/kg CBW	0.05 mg/kg CBW (0.02 mg/kg)	Not available
Foletto M. et al. (2014)*	RCT	Patients with obesity	Laparoscopic bariatric surgery	17/17	Not available	TOF count ≥ 2	2 mg/kg	0.05 mg/kg	Not available

Supplementary Table 2: Characteristics of included trials

RCT: randomized controlled trial; BMI: body mass index; SUG: sugammadex; NEO: neostigmine; ATR: atropine; SD: standard deviations; CBW: corrected body weight; IBW: ideal body weight; TBW: total body weight; LBW: lean body weight; TOF: train-of-four; PTCs: post tetanic counts; *: conference abstract.
