# Supplementary material for: The Efficacy and Adverse Effects of Sugammadex and Neostigmine in Reversing Neuromuscular Blockade Inpatients with Obesity Undergoing Metabolic and Bariatric Surgery: A Systematic Review with Meta-Analysis and Trial Sequential Analysis
Source: Medicina (Kaunas). 2024 Nov 8;60(11):1842. doi: 10.3390/medicina60111842 (PMC11596585; doi:10.3390/medicina60111842)
Supplement: Supplementary file 1 [file medicina-60-01842-s001.zip › Supplementary Materials/Supplementary Material S5 Subgroup analysis including patients undergoing laparoscopic bariatric surgery.pdf]

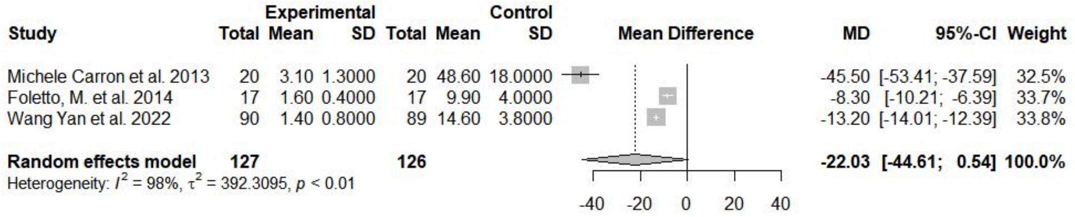

Supplementary Figure 1: Forest plot of the recovery time (min) from administration of the study drug to TOF ratio ≥ 90% (including patients undergoing laparoscopic bariatric surgery); sugammadex (experimental) vs. neostigmine (control).

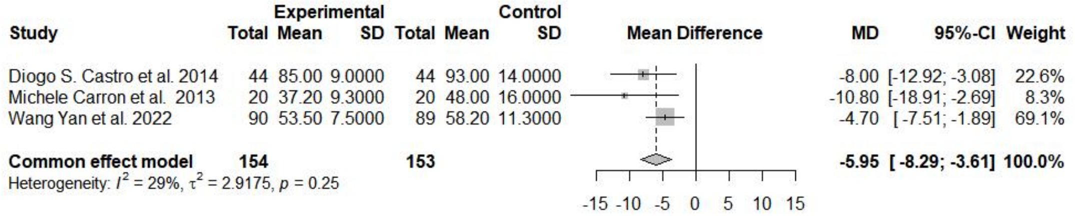

Supplementary Figure 2: Forest plot of the PACU duration (min) (including patients undergoing laparoscopic bariatric surgery); sugammadex (experimental) vs. neostigmine (control).

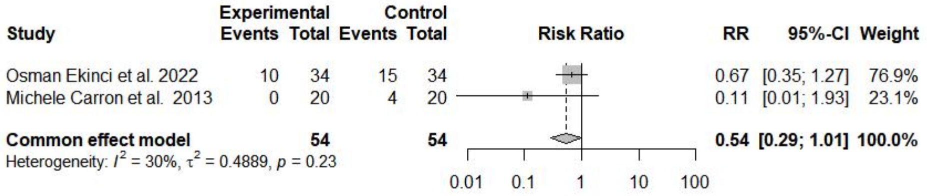

Supplementary Figure 3: Forest plot of the incidence of postoperative cardiovascular complications (including patients undergoing laparoscopic bariatric surgery); sugammadex (experimental) vs. neostigmine (control).
